# *Corynebacteria* in Bovine Quarter Milk Samples—Species and Somatic Cell Counts

**DOI:** 10.3390/pathogens10070831

**Published:** 2021-07-02

**Authors:** Anneke Lücken, Nicole Wente, Yanchao Zhang, Svenja Woudstra, Volker Krömker

**Affiliations:** 1 Department of Microbiology, Faculty of Mechanical and Bioprocess Engineering, University of Applied Sciences and Arts, 30453 Hannover, Germany; anneke.luecken@yahoo.de (A.L.); nicole.wente@hs-hannover.de (N.W.); yanchao.zhang@hs-hannover.de (Y.Z.); 2 Department of Veterinary and Animal Sciences, University of Copenhagen, 1870 Frederiksberg C, Denmark; svenja.woudstra@sund.ku.dk

**Keywords:** bovine mastitis, *Corynebacteria*, SCC, MALDI-TOF MS, species diagnostics

## Abstract

In this species differentiation study of *Corynebacterium* spp. (*C.* spp.), quarter foremilk samples from 48 farms were included. These were obtained from both clinically healthy cows and those with clinical mastitis. First, all samples were examined cyto-microbiologically and all catalase-positive rods were differentiated using the direct transfer method in MALDI-TOF MS. *C. bovis*, *C. amycolatum*, *C. xerosis*, and five other species were identified with proportions of 90.1%, 7.7%, and 0.8% for the named species, respectively, and 1.4% for the remaining unnamed species. In addition, somatic cell count (SCC) was determined by flow cytometry. Based on this, the isolates were classified into four udder health groups: “latent infection”, “subclinical mastitis”, “clinical mastitis” and “others”. Approximately 90% of isolates of *C. bovis* and *C. amycolatum* were from latently and subclinically infected quarters. Of the *C. bovis* isolates, 5.8% were obtained from milk samples from clinical mastitis, whereas *C. amycolatum* was not present in clinical mastitis. The distribution of groups in these two species differed significantly. The geometric mean SCC of all species combined was 76,000 SCC/mL, almost the same as the SCC of *C. bovis*. With 50,000 SCC/mL, the SCC of *C. amycolatum* was slightly below the SCC of *C. bovis*. Through the species-level detection and consideration of SCC performed here, it is apparent that individual species differ in terms of their pathogenicity. Overall, their classification as minor pathogens with an SCC increase is confirmed.

## 1. Introduction

Udder health in the dairy industry is one of the main concerns for the production of high quality food from healthy cows. Economic losses in the dairy industry are mainly caused by subclinical and clinical mastitis due to intramammary infections and are substantially compounded by milk losses [1]. Both from an economic point of view and an animal welfare point of view, mastitis and intramammary infections should be reduced. In order to achieve this goal, the causes must be better understood for prevention. 

As well as typical mastitis pathogens such as *Streptococcus uberis*, *Staphylococcus aureus*, and other major pathogens, other bacteria are also detected in quarter milk samples. Coryneforms are commonly found in bovine milk samples, but their impact on udder health has not been definitively determined. In one region in Germany, a study detected coryneforms in 90% of the farms [2]. Although they are found frequently, they may still be underdiagnosed, possibly due to their slow growth [3]. They can be assessed through a wide range of pathological assessment methods. In some cases, the coryneforms do not even reach the udder tissue but seem to colonize only the teat canal [4]. In addition, there are studies that even suggest they may have a protective effect against major pathogens, stimulating the immune defense by increasing the somatic cell count (SCC) [5]. In other studies, they were detected in subclinical and clinical cases of mastitis and are associated with increased somatic cell count [1,6]. Overall, they are categorized as minor pathogens, similar to non-aureus staphylococci. 

Previous studies usually refer to coryneforms or *Corynebacterium bovis* (*C. bovis*). It is important to note that coryneforms are a group of genera. There are also several *Corynebacterium* spp. (*C.* spp.) in the genus Corynebacteria, which will be the focus of this present study. In routine diagnostics, species differentiation is usually not performed. Species differentiation could be important, however. Staphylococci is also a genus with different species, whereby some individual species in this genus are known to be much more pathogenic than other species. Thus, the question arises whether species differentiation in *C.* spp. can help in the assessment of pathogenicity. 

The aim of this study was to gain an assessment of each Corynebacteria species as a mastitis causing pathogen. For this purpose, quarter foremilk samples were cyto-microbiologically examined in the same way. For samples with a pure culture of coryneforms, the species was determined by matrix-assisted laser desorption/ionization time-of-flight mass spectrometry (MALDI-TOF MS). The SCC of each sample was determined and assigned to the species. Subsequently, the proportions of each species in latently infected quarters and quarters with subclinical and clinical mastitis were investigated. 

## 2. Results

### 2.1. Detected Corynebacterium *spp.* and Their Mastitis Diagnosis

The study included 497 isolates with *C*. spp. A total of eight different species could be identified: *C. bovis*, *C. amycolatum*, *C. xerosis*, *C. confusum*, *C. testudinoris*, *C. frankenforstense*, *C. freneyi*, and *C. kroppenstedtii*. The results of the identified species are shown in Table 1. *C. bovis* accounted for the majority of isolates (90.1%). The second most common one was *C. amycolatum* (7.7%), whereas *C. xerosis* accounted for only 0.8% of the isolates. The remaining species occurred only once or twice and altogether represented only 1.4% of all isolates. Further evaluation mainly considered the three most abundant *C.* spp.

Concerning the ratio of the species *C. amycolatum* to *C. bovis*, the four groups differed significantly from each other (chi-square test *p* < 0.01). Based on the information on cfu/mL, it can be seen that the individual species did not differ with regard to their bacterial excretion.

### 2.2. Group Distribution of Mastitis Status

**Figure 1:**
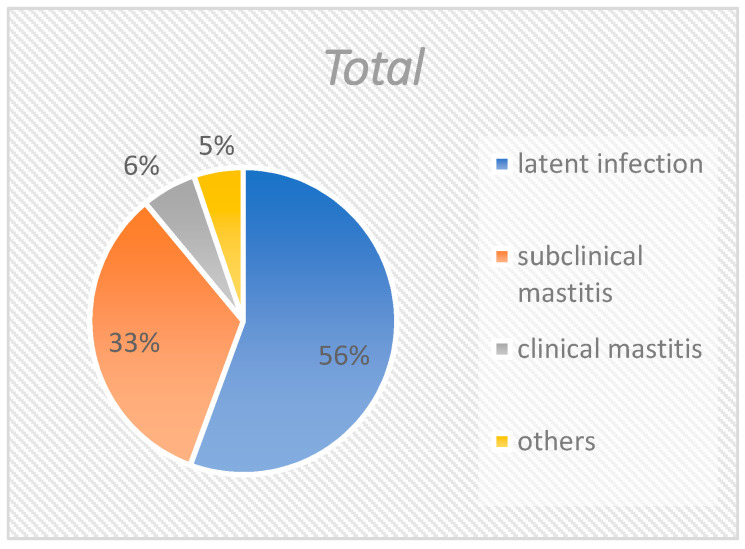
Group distribution of all Corynebacteria isolates. This figure shows the percentage of the different groups in total.

**Figure 2:**
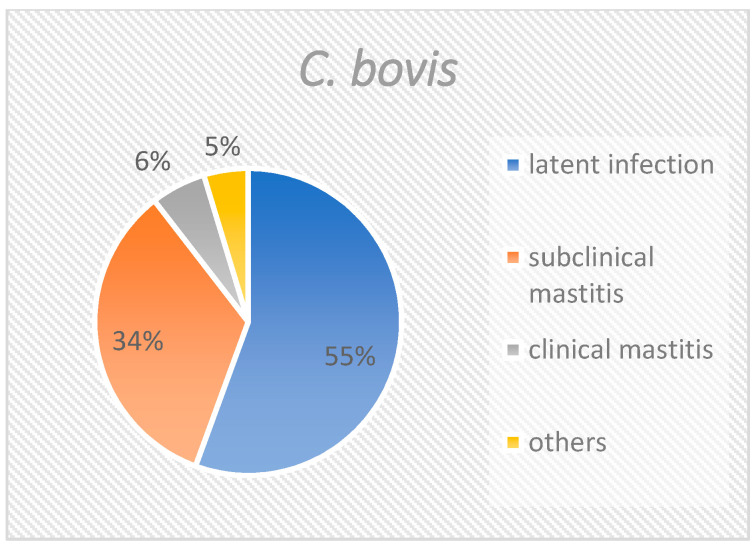
Group distribution of *Corynebacterium bovis*. This figure shows the percentage of the different groups at species level.

**Figure 3:**
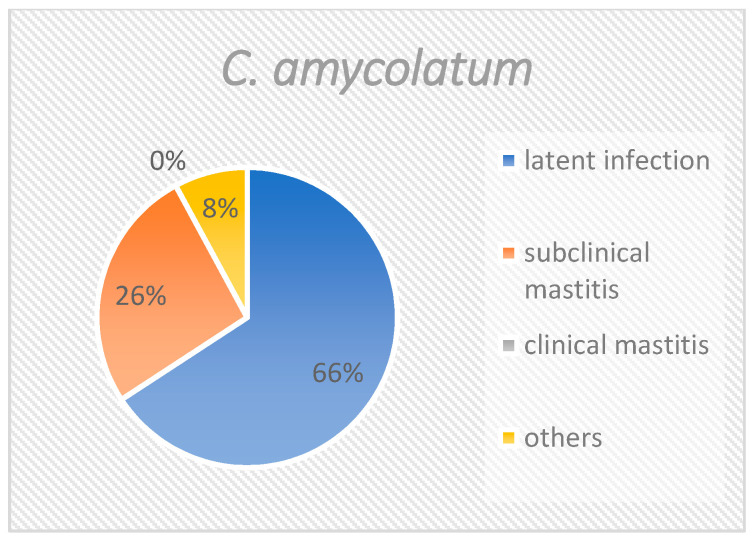
Group distribution of *Corynebacterium amycolatum*. This figure shows the percentage of the different groups at species level.

**Figure 4:**
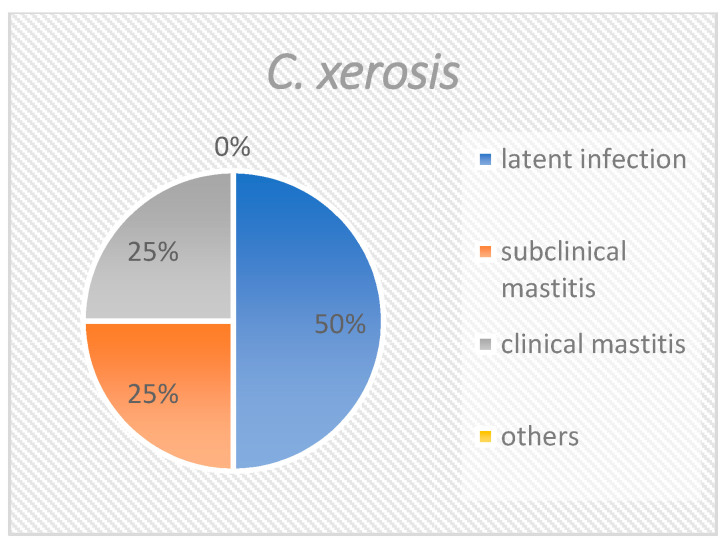
Group distribution of *Corynebacterium xerosis*. This figure shows the percentage of the different groups at species level.

When all isolates are considered together (Figure 1), slightly more than half (55.7%) derived from quarters with latent infections. One third of the corynebacteria (33.4%) were isolated from samples with subclinical mastitis. Approximately 5% of the isolates were from samples with clinical mastitis, and approximately 5% could not be specifically attributed. The distribution of *C. bovis* was similar to the distribution of all isolates (Figure 2), with 55.6%, 33.9%, 5.8%, and 4.7% of isolates being accounted for by the groups “latent infection”, “subclinical mastitis”, “clinical mastitis” and “others”, respectively. *C. amycolatum* could not be isolated from samples of clinical mastitis. Instead, this species was found in 65.8% of latently infected samples and 26.3% of subclinical mastitis samples (Figure 3). Nearly 8% could not be clearly classified. *C. xerosis* could be categorized as “latent infection”, “subclinical mastitis”, and “clinical mastitis” at 50%, 25%, and 25%, respectively (Figure 4).

### 2.3. Species Distribution on Different Farms

The distribution of the species among farms (Table 2) was significantly different at farm level (chi-square test *p* < 0.01). The number of isolates of the different farms varied.

### 2.4. Somatic Cell Count (SCC)

**Figure 5:**
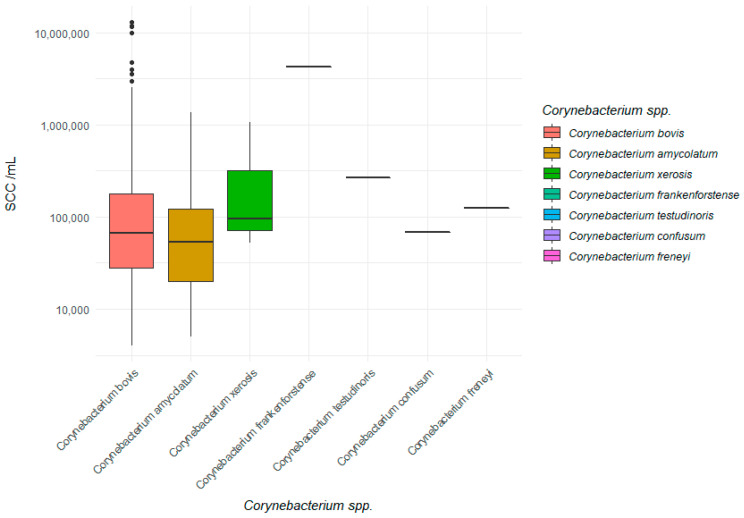
Somatic cell count of different *Corynebacterium* spp.

Looking at the median and geometric mean, the somatic cell count from the foremilk samples in which the SCC could be determined was, simplified, between 50,000 and 100,000 SCC/mL (Table 3). The geometric mean considering all isolates of *C. bovis*, *C. amycolatum,* and *C. xerosis* amounted to 76,000 SCC/mL. While the geometric mean value of *C. amycolatum* with 50,000 SCC/mL was still clearly below the limit of 100,000 SCC/mL, the geometric mean value for *C. bovis* with 78,000 SCC/mL was already closer to the limit of subclinical mastitis. For *C. xerosis*, this value was the highest, reaching 174,000 SCC/mL. The mean somatic cell count of milk samples with *C. bovis* was significantly higher compared to samples with *C. amycolatum* (*p* < 0.05). Figure 5 shows the SCC/mL of each species in a box plot.

## 3. Discussion

The distribution of species shows that *C. bovis* was the most abundant. *C. amycolatum* was also not negligible, with a share of 7.7%. A similar species distribution was found by Watts et al. [7] using 16S rRNA sequencing in *C.* spp. In addition, they were able to identify *C. ammoniagenes* and *C. pseudotuberculosis* several times, which we could not isolate from our samples. Dillmann [8] worked with the Api Coryne system, which also identified *C. bovis* most frequently, but otherwise identified a greater diversity, finding nine Corynebacteria species, three other non-Corynebacteria species, and no *C. amycolatum*. It should be noted that the Api Coryne system identifies some species incorrectly or not at all [7]. Goncalves et al. [9] and Bzdil [10], who sampled cows with mastitis symptoms, worked with MALDI-TOF MS as well and also detected mainly *C. bovis*. *C. amycolatum* occurred only once in the former study and was comparatively abundant in the latter study.

*C. bovis* accounted for the majority of isolates. This explains why the proportions of *C. bovis* in the different groups “latently infected”, “subclinical mastitis”, “clinical mastitis”, and “others” is very similar to the proportions in total. In particular, *C. bovis* showed up in both clinically healthy animals and animals clinically affected by mastitis. Although other *C*. spp. than *C. bovis* were also detected in clinical mastitis, their small number in the present study does not allow further conclusions to be made. The group “others” cannot be clearly evaluated due to missing information on the SCC or with a classification as sensitively changed milk and missing information from the farmer. It is possible that other cases of clinical mastitis were included in this group. Although the species distribution at farm level is significantly different, the presentation of the distribution on the farms may be distorted by the unequal number of samples per farm.

Based on this study, *C. bovis* appears to have a more pathogenic effect on the mammary gland than *C. amycolatum*. *C. bovis* was associated with a greater increase in SCC and, unlike *C. amycolatum*, was detected in clinical mastitis. The study by Bzdil [10], in which only cows with symptoms of mastitis were sampled, *C. bovis* and *C. amycolatum* were detected in 1.65% and 1.02% of all samples, respectively. The proportion of *C. amycolatum* in that previously mentioned study seems to be higher in mastitis samples, but this cannot be assessed with certainty based on our study, since no more reliable statements on prevalence can be made due to the study design. In all studies using the classical milk sampling approach, it is also possible that *Corynebacteria* are secondary colonizers, and that the actual mastitis pathogen is below the limit of detection or could only be detected by another method. Whereas Ericsson et al. [11] sampled only clinical mastitis and found *C.* spp. in less than 1% of samples, Taponen et al. [12] detected *C. bovis* by PCR in 7.9% of cows with mastitis (subclinical and clinical); other species were not studied. It should be noted that the latter study used a different method that also detects dead *Corynebacteria* that may not be related to inflammatory responses. In a study by Tenhagen et al. [2] using only clinically healthy animals, *C. bovis* was identified in a similar range, found in 7.3% of the samples. These were diagnosed by cytobacteriological examination only, yet *C. bovis* was reported as the only coryneform pathogen. Since only clinically healthy animals were involved, it is possible that the detection was either in subclinically diseased animals or that the similar percentages were due to contamination in the same proportion. *C*. spp. are sometimes found only in the teat canal, and thus can lead to positive detection even though they do not colonize the udder. For example, Bexiga et al. [4] were able to reduce the number of positive findings for *C.* spp. by avoiding the teat canal using a teat cannula, but it remains unclear which species was reduced. Based on the samples taken per quarter at the beginning of the milking process, the possibility that the detected *C*. spp. result only from a colonization of the teat canal and not from a latent infection of the udder tissue cannot be excluded [13]. The detection limit was set at 500 cfu/mL or more, as this is standard practice in the laboratory. This ensures that the results do not differ between the different investigators. Since we only chose pure cultures for our study, it is unlikely that contamination came from the teat surface. Otherwise, there would probably be additional microorganisms growing on the blood agar. 

When considering the overall and individual species geometric mean of SCC, the classification of *C.* spp. as minor pathogens is confirmed. For example, Bradley and Green [14] described a value between 50,000 and 150,000 SCC/mL for the SCC of minor pathogens. The geometric mean value for *C. xerosis* alone, however, where only three isolates are examined, is slightly above this limit. Goncalves et al. [6] set the cut-off for the border between minor and major pathogens at 200,000 SCC/mL. Thus, all species fall into the minor pathogen category. In this previously mentioned study, the geometric mean of the SCC was also determined. This was 197,900 and 174,280 SCC/mL for *C.* spp. and *C. bovis*, respectively, and thus appreciably higher than in the present study. However, in the former study, only cows with subclinical mastitis were considered. Here, the definition for subclinical mastitis mainly related to the amount of colony-forming units (>1000 cfu/mL). If the number of cfu/mL was below this amount and the cell count was below 200,000 SCC/mL, one quarter was considered healthy in this aforementioned study. 

The significantly different distribution of udder health groups of *C. bovis* and *C. amycolatum* found in our study suggests that *C. bovis* has a stronger pathogenicity.

### Species Identification

For species differentiation, we used the cut-off level of 1.7 for the MALDI-TOF MS score, as Theel [15] has done previously. The only difference was that in the former study, it referred to *C.* spp. in humans but *C. amycolatum* was also successfully identified there. Another study referred to the cut-off level of 2.0 for the species and considered the isolates with a MALDI-TOF MS score between 1.7 and 2.0 only at genus level [9].

## 4. Materials and Methods

### 4.1. Origin of Samples

The samples for this evaluation came from two sources. Firstly, they originated in part from one farm where the entire dairy herd was sampled at one milking (*n* = 226 *C.* spp. positive milk samples from 116 cows). The remaining 271 isolates came from 47 farms in Germany. Almost all of them were sampled between April 2020 and February 2021. These were all mammary quarter foremilk samples and *C*. spp. was detected in the pure cultures after examination using the same methods. The samples were derived from submissions and projects, and included samples from cows with clinical mastitis, clinically healthy cows, and dry-off samples. A total of 497 isolates were examined.

### 4.2. Cytomicrobiological Examination and Species Differentiation

The samples were analyzed at the Microbiological Laboratory at the Hannover University of Applied Sciences and Arts (Hannover, Germany). Of all samples, 10 µL were spread on one quadrant of an esculin blood agar plate (Oxoid Deutschland GmbH, Wesel, Germany). They were then incubated at 37 °C and examined after 24 and 48 h in accordance with the guidelines of the German Veterinary Association [16]. Grown colonies that showed up as catalase-positive Gram-positive rods in the microbiological examination were identified as coryneforms. Isolates from pure cultures of 500 cfu/mL or more were included in the study. The number of colonies was determined semiquantitatively in three categories: 500 < 1000 cfu/mL, 1000 < 5000 cfu/mL, and ≥5000 cfu/mL. BioTyper™ matrix-assisted laser desorption/ionization time-of-flight mass spectrometry (MALDI-TOF MS; Bruker Daltonik GmbH, Bremen, Germany; Microflex LT/SH smart) was used for further species diagnostics. A direct transfer method without acid extraction was applied. The identification was carried out using the MBT Compass Library (Revision F, MBT 8468 MSP Library). The cut-off for species level was reduced from 2.0 to ≥1.7, in accordance with Theel et al. [15]. The isolates were preserved at −80 °C with 800 µL of brain-heart broth (Merck KGaA, Darmstadt, Germany) and 200 µL of glycerol, and were regrown on esculin blood agar prior to species identification so that they could be examined together. 

### 4.3. Somatic Cell Count (SCC) and Definitions

SCC was measured at quarter level and assessed by flow cytometry (SomaScope Smart, Delta Instruments B.V., Drachten, the Netherlands). Based on somatic cell count and information on clinical mastitis concerning the milk samples, isolates were classified into four groups according to the definition of the German Veterinary Association [13]. Quarters with an SCC < 100,000 SCC/mL were considered latently infected if *C.* spp. was detected in the sample. This means that microorganisms were detected, but there were no symptoms of inflammation. Samples with an SCC of >100,000 SCC/mL and the occurrence of *C.* spp. in the cyto-microbiological examination were considered to have subclinical mastitis since the SCC was elevated but there were no clinical symptoms [13]. Samples from quarters showing clinical symptoms of mastitis, such as milk clots and swelling of the udder tissue, were assigned to the clinical mastitis group. The classification of mastitis was made by the submitting veterinarian or farmer and was indicated on the entry form. The fourth group included samples for which the cell count was missing or the milk was abnormal in the laboratory, but there was no evidence of clinical mastitis had been registered on the sample sheet.

### 4.4. Statistical Analysis

The collection and processing of data were carried out with Microsoft excel (Microsoft Corp., 2010). For analyzing the dataset, the program SPSS 26.0 (IBM Inc., Armonk, NY, USA) was used with quarter milk samples considered as the statistical unit. Associations between Corynebacteria species and herd, SCC, shedding intensity and mastitis diagnosis were examined in univariable analysis (X^2^-test/ANOVA). A *p*-value <0.05 was considered indicative of a statistically significant difference.

## 5. Conclusions

*C. bovis* was found to be the most abundant species of *C.* spp. but *C. amycolatum* also occurred in a non-negligible proportion, among six other less abundant species. The present study shows a stronger pathogenicity of *C. bovis* than *C. amycolatum* and confirms their assessment as minor pathogens leading to an increase in somatic cell count. Some of *C*. spp. could be detected in quarters with clinical mastitis. The detection of different species and different pathogenicity estimates make species diagnostics useful in the presence of coryneforms.

## Figures and Tables

**Table 1:** Distribution of detected *Corynebacteria* spp. according to mastitis diagnosis ^a^.

Species	No. of Isolates (% of Total Isolates Number)	No. of Isolates from Latent Infections (% of All Isolates within One Species)	No. of Isolates from Subclinical Mastitis (% of All Isolates within One Species)	No. of Isolates from Clinical Mastitis (% of All Isolates within One Species)	No. of Other Isolates (% of All Isolates within One Species)
*C. bovis*	448 (90.1) 94/203/145 ^b^	249 (55.6) 46/101/99	152 (33.9) 30/80/39	26 (5.8) 13/11/2	21 (4.7) 5/11/5
*C. amycolatum*	38 (7.7) 6/16/16	25 (65.8) 5/7/13	10 (26.3) 1/6/3	-	3 (7.9) -/3/-
*C. confusum*	2 (0.4) 1/1/-	1 (50) 1/-/-	-	1 (50) -/1/-	-
*C. frankenforstense*	1 (0.2) -/1/-	-	1 (100) -/1/-	-	-
*C. freneyi*	1 (0.2) 1/-/-	-	1 (100) 1/-/-	-	-
*C. kroppenstedtii*	1 (0.2) -/1/-	-	-	-	1 (100) -/1/-
*C. testudinoris*	2 (0.4) 1/1/-	-	1 (50) -/1/-	1 (50) 1/-/-	-
*C. xerosis*	4 (0.8) 3/1/-	2 2/1/-	1 (25) 1/-/-	1 1/-/-	-
Total	497 (100)	277 (55.7)	166 (33.4)	29 (5.8)	26 (5.2)

^a^ This table shows the number of species detected and the percentage of the total isolates they accounted for. In addition, the proportions of each species in the groups “latent infection”, “subclinical mastitis”, “clinical mastitis”, and “others” are presented. *Definitions:* latent infection = *C.* sp. and SCC < 100,000 SCC/mL; subclinical mastitis = *C.* sp. and SCC ≥ 100,000 SCC/mL; clinical mastitis = *C*. sp. and symptoms of clinical mastitis; others = *C.* sp. and SCC not performed. The second line of each species also lists the number of isolates according to the shedding intensity (semi-quantitative abundances: 500 < 1000/1000 < 5000/≥5000 (cfu/mL)). ^b^ In six cases, the semiquantitative number of isolates is missing.

**Table 2:** *Corynebacterium* spp. (*C*. spp.) on farm level. This table shows the number of farms where the listed species occurred.

Present Species	Number of Farms
*C. bovis*	30
*C. amycolatum*	5
*C. xerosis*	2
*C. bovis* and *C. amycolatum*	3
*C. bovis* and *C. xerosis*	1
*C. bovis* and *C. amycolatum* and other species	4
Other species than *C. bovis*	3

**Table 3:** Somatic cell count—median and geometric mean. Median and geometric mean of SSC of the groups latent infection and subclinical mastitis.

Species	Median_SCC/mL	Median_Log(SCC)	GeoMean_SCC	GeoMean_Log(SCC)
*Corynebacterium bovis* (*n* = 401)	66,000	4.82	78,000	4.69
*Corynebacterium amycolatum* (*n* = 35)	53,000	4.72	50,000	4.66
*Corynebacterium xerosis* (*n* = 3)	96,000	4.98	174,000	5.21
Total (*n* = 443) ^a^	66,000	4.82	76,000	4.84

^a^ This item also includes isolates of other species that are less abundant.

## Data Availability

The data presented in this study are available on request from the corresponding author. The data are not publicly available due to agreements with animal owners.

## References

[1] Heikkilä A.-M., Liski E., Pyörälä S., Taponen S. (2018). Pathogen-specific production losses in bovine mastitis. J. Dairy Sci..

[2] Tenhagen B.-A., Köster G., Wallmann J., Heuwieser W. (2006). Prevalence of Mastitis Pathogens and Their Resistance against Antimicrobial Agents in Dairy Cows in Brandenburg, Germany. J. Dairy Sci..

[3] Krömker V., Paduch J.H., Klocke D., Zinke C. (2010). Microbiological investigation of culture negative milk samples of clinical mastitis cows. Milchwissenschaft.

[4] Bexiga R., Pereira H., Pereira O., Leitão A., Carneiro C., Ellis K.A., Vilela C.L. (2011). Observed reduction in recovery of *Corynebacterium* spp. from bovine milk samples by use of a teat cannula. J. Dairy Res..

[5] Lam T.J., Schukken Y.H., van Vliet J.H., Grommers F.J., Tielen M.J., Brand A. (1997). Effect of natural infection with minor pathogens on susceptibility to natural infection with major pathogens in the bovine mammary gland. Am. J. Vet. Res..

[6] Gonçalves J.L., Tomazi T., Barreiro J.R., Beuron D.C., Arcari M.A., Lee S.H.I., Martins C.M.d.M.R., Araújo Junior J.P., dos Santos M.V. (2016). Effects of bovine subclinical mastitis caused by *Corynebacterium* spp. on somatic cell count, milk yield and composition by comparing contralateral quarters. Vet. J..

[7] Watts J.L., Lowery D.E., Teel J.F., Rossbach S. (2000). Identification of *Corynebacterium bovis* and other Coryneforms Isolated from Bovine Mammary Glands. J. Dairy Sci..

[8] Dillmann T. (2014). Beziehungen zwischen dem Nachweis von coryneformen Bakterien und Entzündungsparametern in Viertelanfangsgemelkproben von Milchkühen.

[9] Gonçalves J.L., Tomazi T., Barreiro J.R., Braga P.A.d.C., Ferreira C.R., Araújo Junior J.P., Eberlin M.N., dos Santos M.V. (2014). Identification of *Corynebacterium* spp. isolated from bovine intramammary infections by matrix-assisted laser desorption ionization-time of flight mass spectrometry. Vet. Microbiol..

[10] Bzdil J. (2017). Corynebacteria and coryneform microorganisms isolated from bovine milk with symptomps of mastitis. Veterinářství.

[11] Ericsson Unnerstad H., Lindberg A., Persson Waller K., Ekman T., Artursson K., Nilsson-Ost M., Bengtsson B. (2009). Microbial aetiology of acute clinical mastitis and agent-specific risk factors. Vet. Microbiol..

[12] Taponen S., Liski E., Heikkilä A.-M., Pyörälä S. (2017). Factors associated with intramammary infection in dairy cows caused by coagulase-negative staphylococci, *Staphylococcus aureus*, *Streptococcus uberis*, *Streptococcus dysgalactiae*, *Corynebacterium bovis*, or *Escherichia coli*. J. Dairy Sci..

[13] German Veterinary Association (2002). Leitlinien zur Bekämpfung der Mastitis des Rindes als Bestandsproblem.

[14] Bradley A., Green M. (2005). Use and interpretation of somatic cell count data in dairy cows. Practice.

[15] Theel E.S., Schmitt B.H., Hall L., Cunningham S.A., Walchak R.C., Patel R., Wengenack N.L. (2012). Formic acid-based direct, on-plate testing of yeast and *Corynebacterium* species by Bruker Biotyper matrix-assisted laser desorption ionization-time of flight mass spectrometry. J. Clin. Microbiol..

[16] German Veterinary Association (2018). Leitlinien der Labordiagnostik der Mastitis—Probennahme und Mikrobiologische Untersuchung.

